# Breed-Specific Skull Morphology Reveals Insights into Canine Optic Chiasm Positioning and Orbital Structure through 3D CT Scan Analysis

**DOI:** 10.3390/ani14020197

**Published:** 2024-01-07

**Authors:** Yoichiro Ichikawa, Nobuyuki Kanemaki, Kazutaka Kanai

**Affiliations:** 1 Department of Small Animal Internal Medicine II, School of Veterinary Medicine, Kitasato University, 35-1 Higashi 23 Ban-Cho, Towada 034-8628, Aomori, Japan; yoichiro@mwc.biglobe.ne.jp; 2 Ichikawa Animal Hospital, 118-3 Negiuchi, Matsudo-shi 270-0011, Chiba, Japan; 3 School of Veterinary Medicine, Azabu University, 1-17-71 Fuchinobe, Chuou-ku, Sagamihara 252-5201, Kanagawa, Japan; kanemaki@azabu-u.ac.jp; 4 DVMs Animal Medical Center Yokohama, 2-2 Sawatari, Kanagawa-ku, Yokohama 221-0844, Kanagawa, Japan

**Keywords:** optic chiasm, orbital shape, dogs, skull index, brachycephalic, mesocephalic, dolichocephalic, computed tomography, landmark-based morphological analysis

## Abstract

**Simple Summary:**

Short-headed dogs exhibit shallow orbits and forward-facing eyes, while their medium- and long-headed counterparts have deep orbits with relatively laterally oriented eyes; these traits are classified by skull index (SI) value. In this study, we employed landmark-based morphometric analysis based on computed tomography scan data of 50 adult dogs to investigate the correlation between the SI and optic chiasm, and orbital shape. We found a consistent placement of the optic chiasm at the anterior neurocranial margin across all breeds. However, short-headed breeds exhibit a wider angle between the bilateral optic canals, and the anterior margin of the zygomatic bone-forming orbit was wider in the anterior direction compared to medium- and long-headed breeds. Breed-specific orbital differences were determined by the zygomatic bone, which connects the face to the neurocranium. The orbital margin of the zygomatic bone projects outward and forward, correlating with the degree of facial shortening. Taken together, our findings suggest that the zygomatic bone influences breed-specific orbital formation, especially in cases of facial shortening.

**Abstract:**

This study’s CT scan-based morphometric analysis of 50 adult dogs explored the relationship between skull shape variations (determined by the skull index, SI), optic chiasm, optic canals, and orbital shape. Dogs were classified as brachycephalic (SI ≥ 59), mesocephalic (SI ≥ 51 but <59), and dolichocephalic (SI < 51). No significant age or weight differences were observed. Skull lengths (brachycephalic: 11.39 ± 1.76 cm, mesocephalic: 15.00 ± 2.96 cm, dolichocephalic: 17.96 ± 3.44 cm) and facial lengths (brachycephalic: 3.63 ± 1.00 cm, mesocephalic: 6.46 ± 1.55 cm, dolichocephalic: 8.23 ± 1.03 cm) varied significantly, with shorter orbital depths (brachycephalic: 2.58 ± 0.42 cm, mesocephalic: 3.19 ± 0.65 cm, dolichocephalic: 3.61 ± 0.77 cm) in brachycephalic dogs. The optic chiasm-to-inion horizontal length ratio to cranial horizontal length positively correlated with the SI (r = 0.883, *p* < 0.001), while the ratio to neurocranial length showed no SI correlation (range: 55.5–75.0). Brachycephalic breeds had a significantly wider optic canal angle (93.74 ± 16.00°), along with broader lacrimal-zygomatic and zygomatic frontal process angles. These findings highlight the zygomatic bone’s role in influencing breed-specific orbital variations by connecting the face to the neurocranium, projecting the orbital rim outward and forward with facial shortening.

## 1. Introduction

The positioning of the optic chiasm is associated with the orbital shape. Understanding how alterations in the configuration of the orbit influence the optic chiasm’s location is fundamental for comprehending visual pathways in animals. Specifically, variations in orbital shape, often associated with distinct dog breeds, play a crucial role in shaping the optic chiasm’s position within the skull. The optic chiasm is located at the optic chiasm groove where the optic nerves intersect in the anterior inferior part of the brainstem [1]. The optic chiasm groove marks the point where the left and right optic nerves cross the optic canals in the skull, and the optic chiasm is near the orbit. The optic chiasm’s proximity to the orbit emphasizes its position, directly influenced by the orbital shape of the skull.

Dog skulls are classified by breed into three groups based on their morphology: brachycephalic, mesocephalic, and dolichocephalic [1]. There are inherent shape variations ranging from dolichocephalic breeds with elongated skulls to brachycephalic breeds with broader skulls [2,3]. Craniogenesis is influenced by gene expression [4,5,6]. The skull shape influences the form of the orbit, where the eyeballs are positioned. Brachycephalic dogs exhibit a shallower orbit compared to dolichocephalic breeds, which is attributed to the retraction and formation of the maxilla and nasal bone. This results in a facial structure characterized by a shallow orbit, more prominent front-facing eyeballs, and nasal folds. The short anterior–posterior length of the brachycephalic skull, the shallow orbit, and the wide orbital width contribute to differences in visual function and behavior compared to the dolichocephalic breeds [7,8,9].

Dog skull bone samples have been studied to analyze endemic breeds and understand breed-specific features. However, more recently, computed tomography (CT) data of the cranial region have been used. This approach not only allows the examination of the external shape of the skull but also facilitates the analysis of breed differences in the internal structures of the skull [10,11,12,13,14,15]. Research on skull shape variation has extensively explored structural variation between the face and neurocranium, examined metrological changes in facial landmarks, and investigated internal structures within the neurocranium. In recent decades, computed tomography (CT), magnetic resonance imaging (MRI) and, to a lesser extent, nuclear medicine (NM) have been increasingly used in veterinary clinical practice [16]. Therefore, imaging evaluation of the eye and its environment in the management of periocular and oral surgical tumors in dogs and cats is being considered [17,18,19]. The optic pathway has also been studied under in vivo conditions in dogs and cats [20,21]. However, there is a notable absence of metrological studies of orbital landmarks and the location of the optic chiasm within the skull.

In this study, canine CT data were utilized to construct a 3D model of the skull. The impact of breed-specific variations in skull shape on both optic chiasm position and orbital rim landmarks was examined. This analysis employed a landmark-based morphometric analysis using the structural location of the skull as the reference index.

## 2. Materials and Methods

### 2.1. Subjects

Fifty dogs (weight: 10.7 ± 10.81 kg, age: 8.7 ± 3.08 years) were randomly selected from dogs that presented at Ichikawa Animal Hospital with various diseases, required CT imaging, and had no abnormal head CT findings. The selected breeds included Miniature Dachshund, Toy Poodle, American Cocker Spaniel, Cavalier King Charles Spaniel, French Bulldog, Yorkshire Terrier, Miniature Schnauzer, Pembroke Welsh Corgi, Shetland Sheepdog, Shih Tzu, Jack Russell Terrier, Shiba Inu, Pomeranian, Bernese Mountain Dog, Border Collie, Golden Retriever, Labrador Retriever, and Great Dane.

### 2.2. Preparation

Dogs underwent approximately 16 h of fasting.

### 2.3. Sedation and Anesthesia Induction

Dogs were sedated with 30 μg/kg atropine (atropine sulfate; Mitsubishi Tanabe Pharma Co., Osaka, Japan), 0.15–0.2 mg/kg butorphanol (Betorufaru; Meiji Seika Pharma Co, Ltd., Osaka, Japan) and 0.2 mg/kg atropine and 0.1–0.15 mg/kg midazolam (Dormicum, Astellas Pharma, Tokyo, Japan) were administered intravenously. Anesthesia was induced with 6 mg/kg propofol (Fresenius Kabi, Tokyo, Japan), followed by tracheal intubation. Maintenance of anesthesia was achieved with isoflurane (Isoflu; DS Pharma Animal Health, Osaka, Japan) and oxygen. The oxygen was administered at a flow rate of 1–2 L/min, and the minimum alveolar concentration of isoflurane was regulated between 1.5 and 2.0.

### 2.4. CT Scanning Techniques

Dogs were positioned in sternal recumbency on a CT bed, using straps and foam rollers for stability.

All CT scans were conducted with a 160-slice scanner (160MSCT: Aquilion Lightning TSX-036A; Canon Medical Systems, Otawara, Japan). Imaging was performed using 80 × 0.5 mm collimation, 0.5 mm reconstructed slice thickness, 0.813 pitch, 0.75 s per rotation, automatic output of 100–300 mA/120 kV, 780 mm gantry aperture, and a 512 × 512 matrix with case-specific imaging distance.

Some dogs received intravenous contrast during anesthesia, but these data were utilized for the analysis of pre-contrast CT scans.

### 2.5. Image Analysis and Measurement Methods of Angles, Lengths, and Vertical Lengths

The CT images were processed using DICOM processing software (OsiriX MD v.13.0.3, Pixmeo SAR, Bernex, Switzerland) with a window length of 350 Haunsfield units (HU) and a window width of 2000 HU for reconstructing canine skulls [10,22,23]. Individual 3D models of the skulls were generated using the default mode of the surface-rendering function in OsiriX MD. Landmarks essential for measurements were assigned using the point function of the Region of Interest (ROI). The 3D coordinates of the assigned landmarks were first pointed at the landmarks three times in 3D viewer mode, the three-point coordinates were obtained in 2D viewer mode, and the average coordinates were recorded as 3D coordinate data. All measurements on CT images were conducted by two operators (YI and NK) with a double-checking procedure.

**Figure 1:**
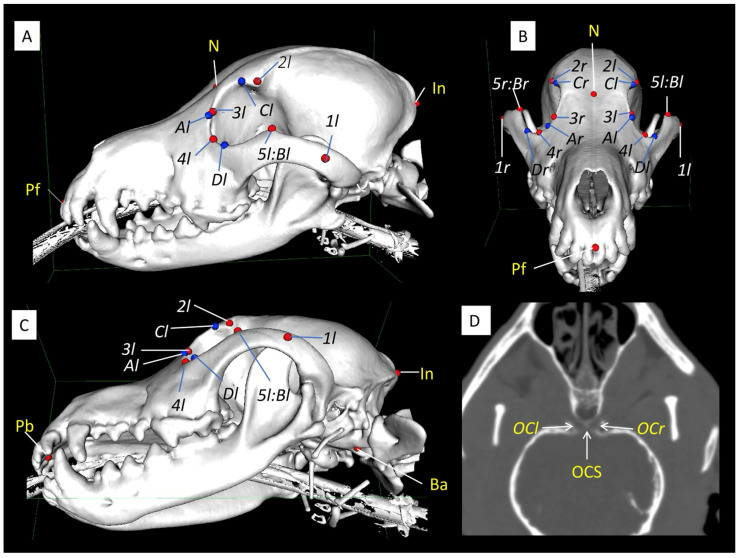
Skull landmarks. (**A**): Left lateral view; (**B**): frontal view; (**C**): left ventral lateral view of the 3D skull model; (**D**): paracoronal CT image showing the optic canal and optic chiasmatic groove of the skull. Landmarks: prosthion_front; Pf, prosthion_back; Pb, nasion; N, inion; I, basion; Ba, zygomaticomaxillary point; *1l* and *1r,* frontozygomatic process; *2l* and *2r*, the orbital rim of frontolacrimal junction; *3l* and *3r*, the orbital rim of lacrimal–zygomatic junction; *4l* and *4r*, zygomatic frontal process; *5l:Bl* and *5r:Br*, orbital horizontal points; A (*Al* and *Ar*) and B (*5l:Bl* and *5r:Br*), orbital vertical points; C (*Cl* and *Cr*) and D (*Dl* and *Dr*) in the A, B and C views, and optic chiasmatic sulcus; OCS (where the optic chiasm is located) and optic canal; OC (*OCl* and *OCr*) in the D view. See Appendix A for an anatomical explanation of terms [26].

The landmarks employed in this study are illustrated in Figure 1 (refer to Appendix A for anatomical descriptions). The landmarks are categorized into three groups. The first group consists of indicators characterizing the skull: the prosthion (front and back), the nasion, the inion, the basion, and the zygomandibular point. The second group comprises indicators related to the orbital margin: the frontozygomatic process, the orbital rim of the frontal–lacrimal junction, the orbital rim of the lacrimal–zygomatic junction, the frontal zygomatic process, the orbital horizontal points, and the orbital vertical points. The third group involves the optic chiasm and the optic canals [1,24]. The coordinates of the landmarks in the third group were obtained from the video image in the paracoronal image (Figure 1D), while those of the remaining landmarks were derived from the 3D skull model (Figure 1A–C). The 3D image data of the landmarks marked on the 3D skull model were exported as STL data and processed using the Hollow Tool with Autodesk Meshmixer (freeware, http://www.meshmixer.com (accessed on 24 May 2023)). The images facilitated the examination of the spatial relationship between the optic chiasmatic sulcus and other landmarks. These landmarks were incorporated as points into the 3D skull model surface-rendering function of the 3D viewer. Subsequently, calculations were performed, including the distance between the two points, the horizontal distance to the points, and the angle with the optic chiasm as the vertex (refer to Appendix B for the calculation tool and formula). The measurements used in this study included a range of parameters: Skull length, skull width, skull base length, skull index (SI), facial length, neurocranial length, skull horizontal length, optic chiasm-to-inion horizontal length, frontal length, lacrimal bone length, palatal length, orbital vertical length, orbital horizontal width, orbital depth, orbital index, orbital area, zygomatic process angle, frontal–lacrimal angle, lacrimal–zygomatic angle, frontal process angle, and optic chiasm angle (refer to Appendix C for an overview of these measurements) [1,25,26]. Measurements were computed using spreadsheet software (Microsoft Excel v16.77.1; Microsoft Corporation, Tokyo, Japan).

### 2.6. Statistics

Statistical analyses employed SPSS Statistics (version 29.0.1.0, IBM Corp, Armonk, New York, NY, USA). Group comparisons, including age, weight, and all measured and calculated values are presented as means and standard deviations. For between-group comparisons, multiple comparison tests were used for items for which significance was found in the F-values of the one-way analysis of variance results. The Scheffe test was used for multiple comparisons assuming equal variances, and Tamhane’s T2 test when variances were not assumed to be equal. Pearson’s analysis was employed for bivariate correlations, with all significance levels set at 0.05.

## 3. Results

### 3.1. Skull Characteristics

The 3D skull model derived from the CT data included 50 specimens classified by SI, based on the classification by Czeibert [27], into 13 brachycephalic dogs with a SI of 59 or greater, 31 mesocephalic dogs with a SI of 51 or greater and less than 59, and six dolichocephalic dogs with a SI less than 51. Table 1 presents the measured distances between skull and orbital landmarks, along with the corresponding indices for these three groups. No significant differences were observed in age or weight among the three groups. The only significant differences in skull shape among the three groups were found in the skull index (SI) and face length: 76.04 ± 11.65 and 3.63 ± 1.00 cm in brachycephalic, 56.25 ± 2.23 and 6.46 ± 1.55 cm in mesocephalic and 48.72 ± 1.33 and 8.23 ± 1.03 cm in dolichocephalic dogs, respectively. Conversely, the aspects demonstrating significant differences between the brachycephalic group and the mesocephalic and dolichocephalic groups were skull length (11.39 ± 1.76 cm in brachycephalic, 15.00 ± 2.96 cm in mesocephalic and 17.96 ± 3.44 cm in dolichocephalic dogs), skull base length, and horizontal skull length. In terms of items reflecting the orbital rim shape, significant differences between the brachycephalic group and the mesocephalic and dolichocephalic groups were observed in frontal length and orbital depth: 2.2 ± 0.37 and 2.58 ± 0.42 cm in brachycephalic, 2.41 ± 0.30 and 3.19 ± 0.65 cm in mesocephalic and 2.59 ± 0.11 and 3.61 ± 0.77 cm in dolichocephalic dogs, respectively. However, no significant differences were found in lacrimal length and malar length, which constitute the anterior outer surface of the orbit. No significant differences were identified among the three groups in neurocranial length or chiasma–inion horizontal length. Additionally, there were no distinctions in orbital area and orbital index between the three groups. These findings were classified according to the SI by the sample we used, which showed that brachycephalic dogs were shorter in skull length and orbital depth than mesocephalic and dolichocephalic dogs.

### 3.2. Optic Chiasm Position

**Figure 2:**
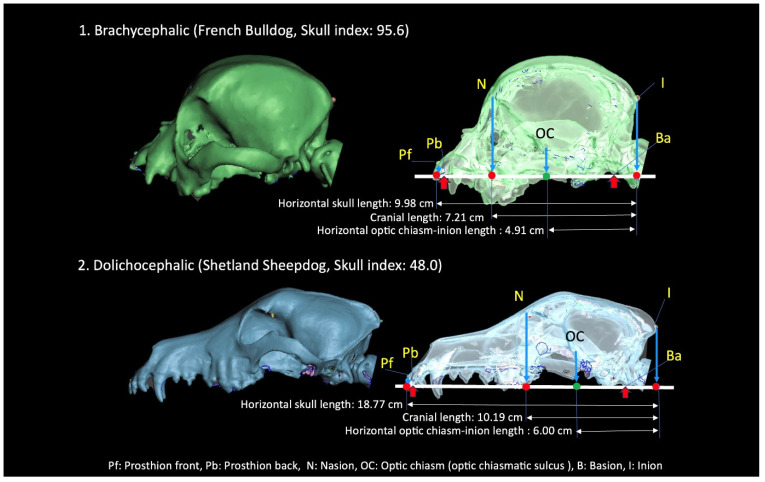
Images of the location of the front prosthion, the nasion, the optic chiasm, and the inion in a brachycephalic and a dolichocephalic dog.

Due to the location of the anterior aspect of the orbit at the facial/neurocranial junction, the optic chiasm’s position on the long axis of the skull was examined at this junction in both brachycephalic and dolichocephalic breeds, one with a SI of 95.6 and one with a SI of 48.0 (Figure 2). The horizontal lengths from the optic chiasm–inion lengths in the brachycephalic and dolichocephalic breeds were measured at 4.91 cm and 6.00 cm, respectively. In contrast, when measured from the inion, the horizontal skull and neurocranial lengths were 9.98 cm and 7.21 cm, respectively, in the brachycephalic dog and 18.77 cm and 10.19 cm in the dolichocephalic dog.

**Figure 3:**
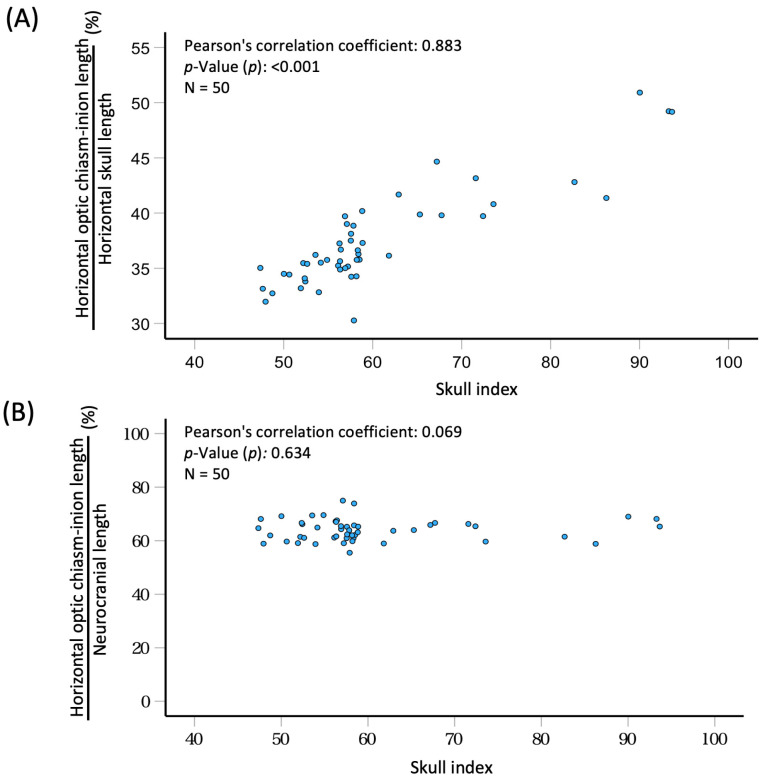
Ratio of horizontal optic chiasm–inion length to horizontal skull length (**A**) or neurocranial length (**B**) versus skull index.

Given that the difference in horizontal skull length between these two breeds exceeds the difference in neurocranial length, it is crucial to consider both horizontal skull length and neurocranial length when assessing breed-related differences in the position of the optic chiasm along the long cranial axis. Consequently, we investigated the correlation between the SI and the position of the optic chiasm, examining both horizontal skull length and neurocranial length across all 50 dogs, using the optic chiasm–inion horizontal length as the criterion (Figure 3). The ratio of the optic chiasm to the horizontal skull length exhibited a positive correlation with the SI (r = 0.883, *p* < 0.001). However, the ratio of horizontal optic chiasm–inion length to neurocranial length did not show a correlation with the SI. The range of ratios spanned from 55.5 to 75.0, with a mean ratio of 64.1 ± 4.01%.

### 3.3. Angle Measurements

**Figure 4:**
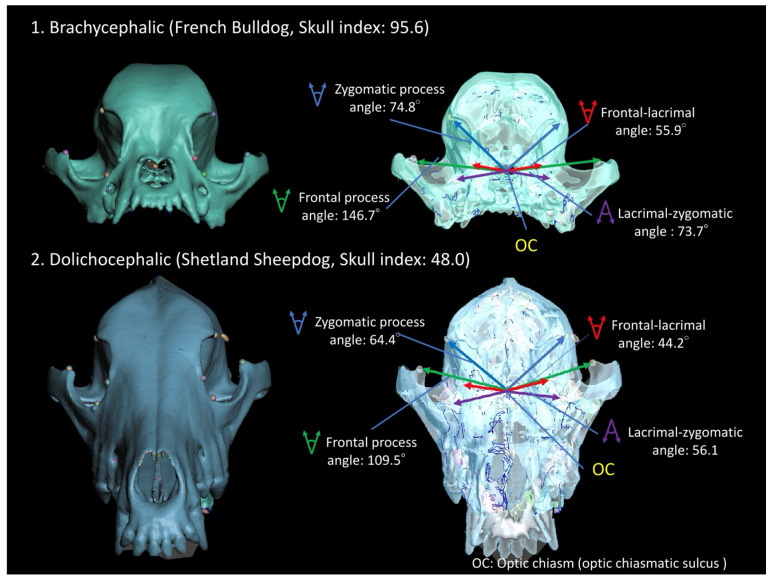
3D positional images illustrating the measured angles of the orbital rim landmarks with the optic chiasm at the apex in (1) brachycephalic and (2) dolichocephalic dogs as depicted in Figure 2.

In contrast, the optic chiasm serves as the convergence point for the visual information from the left and right eyeballs within the neurocranium, acting as a crucial landmark for elucidating the orbital arrangement that houses the eyeballs. Consequently, when assessing the angle between the left and right orbital rim landmarks in the brachycephalic and dolichocephalic dogs featured in Figure 2, with the optic chiasm located rostrally at the ventral surface of the diencephalon in the anterior fossa, caudal to the jugum sphenoidale, it was observed that the angle between the outer orbital rim landmarks was greater in the brachycephalic dog compared to the dolichocephalic dog (Figure 4). Consequently, an examination of the three SI groups was conducted (Table 2).

The lacrimal–zygomatic and frontal process angles of the brachycephalic group exhibited significantly greater widths compared to both mesocephalic and dolichocephalic groups. Furthermore, the optic canal angle (93.74 ± 16.00°) within the brachycephalic group was significantly broader than that of the mesocephalic (67.87 ± 10.76°) and dolichocephalic (61.05 ± 11.02°) groups, along with the lacrimal–zygomatic and frontal process angles.

## 4. Discussion

In this study, we used landmarks from a 3D skull model generated from CT data to establish the metrological basis for the shortened facial features of brachycephalic breeds compared to mesocephalic and dolichocephalic breeds. Dogs are categorized into three groups based on cranial proportions: brachycephalic (short and broad), dolichocephalic (long and narrow), and mesocephalic (intermediate proportions). The dolichocephalic and mesocephalic breeds can be collectively termed the normocephalic breeds, particularly in contrast to the brachycephalic breeds [28]. Consistent with earlier findings, the facial features of the brachycephalic breeds exhibit a shortened morphology compared to the mesocephalic and dolichocephalic breeds [6,7,29,30]. In brachycephalic breeds, the rostrum’s proximal end shortening leads to a widened hard palate, a shortened presphenoid with a consequent shortening of the presphenoid and associated reduction of the rostral cranial fossa, and dorsal rotation of the neurocranium [3,10,31,32]. The optic chiasm positioned at the anterior border of the rostral cranial fossa plays a vital role in integrating visual information from the left and right eyes through the optic nerve, facilitating stereopsis and visual perception. According to Selba et al. [32], the optic canal and orbital fissure have a slight outward and forward shift in the brachycephalic species. Nevertheless, the specific relationship between the optic chiasm and orbital landmarks remains unclear.

The traditional approach for measuring the SI, still in use, involves direct skull measurement using a ruler [33,34,35]. Additionally, alternative methods utilize photographic or radiographic images of the head for SI measurement [36,37,38,39,40,41]. The advancement of CT technology has introduced methods to measure the SI from CT data. One approach involves calculating the SI by measuring the cranial length and width from the midsagittal and dorsal plane images, respectively [5,42]. Its drawbacks are that it makes it challenging to identify anatomical landmarks and it is limited in its application to measure other cranial landmarks. Another approach involves measuring cranial length and width from midsagittal plane images and 3D skull models derived from CT data to calculate the SI [43]. In this method, generating a 2D screen of the vertical axis plane may impact measurement accuracy. An alternative method involves calculating the SI by acquiring the 3D coordinate points of landmarks and calculating the cranial length and width in 3D space, a requirement for measuring the SI of a 3D skull model [27,32]. This method has been applied in various studies of geometric morphometry [3,6,30,32,44,45,46,47,48,49]. However, our study used landmark-based morphometrics focusing on orbital and optic chiasm landmarks. In this study, the apex of the optic canal angle is identified as the optic chiasm. The position of the optic chiasm appeared to move anteriorly in the skull. Despite craniofacial shortening, the position of the optic chiasm within the neurocranium appeared largely unaffected by changes in skull shape. The ratio of optic chiasm–inion length to horizontal skull length showed a positive correlation with the skull index, while the ratio of optic chiasm–inion length to horizontal skull length remained relatively constant across different skull indices. This finding indicates that the position of the optic chiasm within the neurocranium is independent of breed variations [10].

In this study, brachycephalic breeds exhibited a shorter frontal length, reflecting a reduced distance between the inner orbit and the outer supraorbital margin, compared to mesocephalic and dolichocephalic breeds. In addition, the study found no significant differences in the lacrimal length and malar length, representing the anterior surface of the lid margin, among brachycephalic, mesocephalic, and dolichocephalic breeds. However, brachycephalic breeds exhibited wider lacrimal–zygomatic and frontal process angles compared to the mesocephalic and dolichocephalic groups, contributing to the anterior projection of the outer orbital margin. The brachycephalic breeds exhibited a shallower orbital depth than that of the mesocephalic and dolichocephalic breeds. The orbital configuration of the brachycephalic breeds resulted in a shorter distance between the inner orbit and the outer supraorbital rim compared to the mesocephalic and dolichocephalic breeds. Additionally, the outer part of the orbital rim of the zygomatic bone protruded anteriorly, facilitating the placement of the eyeball in the shallow orbit. Consequently, the brachycephalic species exhibited a wide angle between the bilateral frontal processes with the optic chiasm at their apex. This zygomatic shape in the brachycephalic species signifies a configuration connecting the shortened facial skeleton to the neurocranium, resulting in an orbit that faces the eyeballs straight ahead [7,50]. Moreover, due to the anterior location of the optic chiasm within the neurocranium, the outwardly widened zygomatic bone in brachycephalic species leads to a shallow orbit and a wide angle of the optic canal.

Changes in the shape of the zygomatic bone, linking facial structure to the neurocranium, impact the orbit and subsequently influence eye positioning, retinal ganglion cell density [7], responsiveness to visual stimuli [8,37], self-grooming and allogrooming [38], and trainability [8]. However, short-nosed breeds are predisposed to various health issues, including short-head obstructive airway syndrome, dental problems, skin conditions, spinal malformations, brain damage, and otitis media. Additionally, they are susceptible to eye diseases such as exposure keratitis, large eyelid fissures, and eyelid entropion [51,52,53,54,55,56,57].

## 5. Conclusions

In conclusion, our study focused on the orbital shape variation among brachycephalic, mesocephalic, and dolichocephalic dog breeds, categorized by their skull shapes. While our findings contribute valuable insights, it is essential to note that the study did not explicitly address the initial hypothesis. Our results, obtained through morphometric analysis of CT scan data and landmarks of the orbit, optic canal, and optic chiasm, consistently place the optic chiasm at the anterior neurocranial border across breeds. However, certain limitations should be acknowledged. The study lacks a detailed exploration of the metrics or measurements obtained from the morphometric analysis, limiting the depth of evidence presented. Additionally, the initial hypothesis of the study is not explicitly stated or addressed in the conclusion, leaving room for clarification in future research. Future investigations could delve into the specific metrics obtained from morphometric analysis, providing a more detailed understanding of the observed variations. Moreover, exploring the functional implications of these anatomical differences could broaden our understanding of the relationships between skull shape, orbital morphology, and canine visual capabilities.

## Figures and Tables

**Table 1:** Skull measurements in brachycephalic, mesocephalic, and dolichocephalic dogs. See Appendix C for anatomical terms used in the measurements.

	Skull Index Groups ^1^			ANOVA *p*-Value (F-Value)
	Brachycephalic (59 ≤ Skull Index)	Mesocephalic (51 ≤ Skull Index < 59)	Dolichocephalic (Skull Index < 51)	
Case Number	13	31	6	
Sex	Male: 7, Female: 6	Male: 14, Female: 17	Male: 4, Female: 2	
Age (year)	8.85 ± 3.29	8.94 ± 2.82	8.50 ± 4.37	
Body weight (Kg)	7.65 ± 3.58	10.26 ± 8.63	19.57 ± 23.42	
Skull length (cm)	11.39 ± 1.76 ^ab2(A)^	15.00 ± 2.96 ^b^	17.96 ± 3.44 ^a^	*p* < 0.001 (F = 13.454)
Skull width (cm)	8.64 ± 1.73	8.43 ± 1.66	8.77 ± 1.85	
Skull base length (cm)	9.55 ± 1.51 ^ab(A)^	12.61 ± 2.45 ^b^	15.15 ± 3.43 ^a^	*p* < 0.001 (F = 13.086)
Skull index (SI)	76.04 ± 11.65 ^ab(B)^	56.25 ± 2.23 ^bc^	48.72 ± 1.33 ^ac^	*p* < 0.001 (F = 59.607)
Face length (cm)	3.63 ± 1.00 ^ab(B)^	6.46 ± 1.55 ^bc^	8.23 ± 1.03 ^ac^	*p* < 0.001 (F = 28.753)
Neurocranial length (cm)	7.29 ± 1.00 ^a(A)^	8.19 ± 1.50	9.39 ± 2.31 ^a^	*p* < 0.022 (F = 4.142)
Horizontal skull length ^3^ (cm)	10.92 ± 1.69 ^ab(A)^	14.65 ± 2.88 ^b^	17.62 ± 3.27 ^a^	*p* < 0.001 (F = 15.006)
Frontal length (cm)	2.2 ± 0.37 ^ab(B)^	2.41 ± 0.30 ^b^	2.59 ± 0.11 ^a^	*p* = 0.027 (F = 3.896)
Lacrimal length (cm)	1.04 ± 0.31	0.94 ± 0.22	1.12 ± 0.57	
Malar length (cm)	2.55 ± 0.36	2.67 ± 0.36	2.88 ± 0.65	
Orbital vertical length (cm)	2.94 ± 0.51	2.85 ± 0.36	2.99 ± 0.72	
Orbital horizontal width (cm)	2.79 ± 0.29	2.83 ± 0.32	2.96 ± 0.55	
Orbital depth (cm)	2.58 ± 0.42 ^ab(A)^	3.19 ± 0.65 ^b^	3.61 ± 0.77 ^a^	*p* = 0.002 (F = 7.054)
Orbital index	96.36 ± 8.80	99.51 ± 5.62	99.98 ± 5.37	
Orbital area (cm^2^)	6.54 ± 1.71	6.41 ± 1.54	7.21 ± 3.44	

^1^ Classified by Czeibert [27]. ^2^ Different letters indicate significant differences by multiple comparisons of either Scheffe ^(A)^ or Tamhane ^(B)^ test (*p* < 0.05), based on the results of the equal variance test. ^3^ Horizontal skull length is the sum of the face and cranial length.

**Table 2:** Angles of orbital landmarks using the optic chiasm as the apex and optic canal angle. See Appendix C for anatomical terms used in the measurements.

	Skull Index Groups ^1^			ANOVA *p*-Value (F-Value)
	Brachycephalic (59 ≤ Skull Index)	Mesocephalic (51 ≤ Skull Index < 59)	Dolichocephalic (Skull Index < 51)	
Case Number	13	31	6	
Zygomatic process angle (°)	72.44 ± 10.38	69.61 ± 9.83	66.71 ± 7.87	
Frontal–lacrimal angle (°)	49.56 ± 7.37	44.51 ± 3.73	44.07 ± 2.94	
Lacrimal–zygomatic angle (°)	65.88 ± 8.45 ^ab2(A)^	57.27 ± 5.83 ^b^	56.26 ± 3.95 ^a^	*p* < 0.001 (F = 8.967)
Frontal process angle (°)	138.87 ± 9.82 ^ab(B)^	124.3 ± 10.38 ^b^	116.03 ± 6.34 ^a^	*p* < 0.001 (F = 14.231)
Optic chiasm angle (°)	93.74 ± 16.00 ^ab(B)^	67.87 ± 10.76 ^b^	61.05 ± 11.02 ^a^	*p* < 0.001 (F = 23.776)

^1^ Classified by Czeibert [27]. ^2^ Different letters indicate significant differences by multiple comparisons of either Tamhane ^(A)^ or Scheffe ^(B)^ test (*p* < 0.05), based on the results of the equal variance test.

## Data Availability

The authors cannot publicly share the data supporting this study for privacy reasons, although may be shared upon reasonable request to the corresponding author if appropriate.

## Appendix A. Landmarks and Abbreviations of Anatomical Descriptions in the Skull

**Landmarks of the Skull**	**Abbreviations Indicated in Figure 1**	**Anatomical Descriptions**
Prosthion, front	Pf	Most anterior (front) point on the alveolar process of the maxilla
Prosthion, back	Pb	Most anterior (back) point on the alveolar process of the maxilla
Nasion	N	Midpoint of the frontonasal suture and the nasofrontal suture
Inion	I	Most prominent point at the external occipital protuberance
Basion	Ba	Anterior margin of the foramen magnum
Zygomaticomaxillary point	*1l*, *1r*	Widest points of the left or right zygomatic arch
Frontozygomatic process	*2l*, *2r*	Left or right zygomatic process of the frontal bone
Orbital rim of the frontolacrimal junction	*3l*, *3r*	Orbital rim of the frontal–lacrimal junction on the left or right side
Orbital rim of the lacrimal-zygomatic junction	*4l*, *3r*	Orbital rim of the lacrimal–zygomatic junction on the left or right side
Zygomatic frontal process	*5l*, *5r*	Frontal process of the zygomatic bone on the left or right side
Optic chiasmatic sulcus	OCS	Groove on the upper surface of the sphenoid bone where the optic chiasm lies
Optic chiasm	OC	Point where the two optic nerves cross
Optic canal	*OCl*, *OCr*	Bony passage located in the sphenoid bone that serves as a conduit for the optic nerve on the left or right side
Orbital horizontal points	A and B	Rostral (A) and caudal (B) margins of the orbital rim to measure the orbital horizontal width; the caudal margin (B) is in the same position as the zygomatic frontal process.
Orbital vertical points	C and D	Supraorbital (C) and infraorbital (D) margins of the orbit rim to measure the orbital vertical length

## Appendix B. Automated Calculation Tools for Calculating Distances, Horizontal Distances, and Angles for 3D Skull Models Obtained from Computed Tomography

Appendix B provides an Excel measurement sheet that performs length, horizontal length, and angle measurements from the 3D CT data in this paper. In this paper, the analysis was performed on an Excel sheet containing formulas using Excel’s macro function.

The length, horizontal length, and angle can be calculated by entering the 3D coordinate data into the Excel sheet in Appendix B.

The calculation method is described below.

Step 1: Open Appendix C. Select “Enable Macro” and open the file in the opening process.

Step 2: In the “Calculate” sheet of Appendix C, select Length, Horizontal Length, and Angle, and press the “Clear” button.

Step 3: Enter the 3D coordinate data you want to calculate.

Step 4: Press the “Calculate” button.

Step 5: The calculation result will be displayed in “Result”.

If you want to measure next, press “Clear”, enter data, and then “Calculate”.

The formulas used as 3D coordinate data in the Excel sheet are as follows.

(1) Length Calculation

The distance between two points |AB| is calculated as the distance between point A (x1, y1, z1) and point B (x2, y2, z2) in 3D space. In other words, $the\;\left|AB\right|=\sqrt{{\left(x2-x1\right)}^{2}+{\left(y2-y1\right)}^{2}+{\left(Z2-z1\right)}^{2}}$

Therefore,

|AB|= SQRT((x2 − x1)^2^ + (y2 − y1)^2^ + (z2 − z1)^2^)

(2) Horizontal Length Calculation

The horizontal lengths are the facial length, the neurocranial length, the horizontal skull length, and the horizontal optic chiasm–inion length. The line segment indicating the length is assumed to be parallel to the line segment of the back prosthion and basion.

Therefore, the horizontal length between points A (x1, y1, z1) and B (x2, y2, z2) was obtained by drawing a line between the back prosthion C (x3, y3, z3) and the basion D (x4, y4, z4) and the perpendicular points E (x5, y5, z5) and F (x6, y6, z6) of points A and B, respectively. The horizontal length between A and B is obtained as |EF|. Under these conditions, the inner product of the two orthogonal vectors is zero. In other words,

$$\overset{⃑}{AE}\cdot \overset{⃑}{CD}=0\;and\;\overset{⃑}{BF}\cdot \overset{⃑}{CD}=0$$

The following equations are derived from the above.

The x5, y5, z5 of the perpendicular point E (x5, y5, z5) of point A (x1, y1, z1) is found as follows.

Q = x4 − x3

R = y4 − y3

S = z4 − z3

Assuming that

$$\left(\begin{array}{c} x5\\ y5\\ z5 \end{array}\right)=\left(\begin{array}{c} x3+Q*((Q*x1+R*y1+S*z1)-(Q*x3+R*y3+S*z3))\;/\;(Q^{2}+R^{2}+S^{2})\\ y3+R*((Q*x1+R*y1+S*z1)-(Q*x3+R*y3+S*z3))\;/\;(Q^{2}+R^{2}+S^{2})\\ z3+S*((Q*x1+R*y1+S*z1)-(Q*x3+R*y3+S*z3))\;/\;(Q^{2}+R^{2}+S^{2}) \end{array}\right)$$

The x6, y6, z6 of the perpendicular point F (x6, y6, z6) at point B (x2, y2, z2) can also be found as follows.

$$\left(\begin{array}{c} x6\\ y6\\ z6 \end{array}\right)=\left(\begin{array}{c} x3+Q*((Q*x2+R*y2+S*z2)-(Q*x3+R*y3+S*z3))\;/\;(Q^{2}+R^{2}+S^{2})\\ y3+R*((Q*x2+R*y2+S*z2)-(Q*x3+R*y3+S*z3))\;/\;(Q^{2}+R^{2}+S^{2})\\ z3+S*((Q*x2+R*y2+S*z2)-(Q*x3+R*y3+S*z3))\;/\;(Q^{2}+R^{2}+S^{2}) \end{array}\right)$$

From this result, the horizontal length of AB,

|EF| = SQRT((x6 − x5)^2^+(y6 − y5)^2^+ (z6 − z5)^2^)

(3) Angle Calculation

The angle with the optic chiasm as the vertex was calculated as the angle between point A (x1, y1, z1) and point B (x2, y2, z2) with the optic chiasm O (x0, y0, z0) as the vertex. Under this condition, the cosine of the angle θ formed by the vectors $\overset{⃑}{OA}$ and $\overset{⃑}{OB}$ is the inner product of the vectors. In other words,

$$\cos\theta =\frac{\overset{⃑}{OA}\cdot \overset{⃑}{OB}}{\left|\overset{⃑}{OA}\right|\left|\overset{⃑}{OB}\right|},0\leq \theta \leq 180{}^{\circ},\;however\;\overset{⃑}{OA}\neq 0\;and\;\overset{⃑}{OB}\neq 0$$

The following equation was derived from the fact that

θ (radians) = ACOS(((x1 − x0) × (x2 − x0) + (y1 − y0) × (y2 − y0) + (z1 − z0) × (z2 − z0))/((SQRT((x1 − x0)^2^ + (y1 − y0)^2^ + (z1 − z0)^2^)) × (SQRT((x2 − x0)^2^ + (y2 − x0)^2^ + (z2 − z0)^2^))))

θ (°; degrees) = θ (radians)* 180/PI()

However, only the optic canal angle was measured directly from the paracoronal image in MPR mode using an angle measurement ROI.

## Appendix C. Skull Measurements and Anatomical Descriptions

**Skull Measurement Items**	**Anatomical Descriptions**
Skull length	Length between the front prosthion and inion
Skull width	Length between the widest point of the zygomatic arches
Skull base length	Length between the back prosthion and basion
Skull index (SI)	Skull width/skull length × 100
Face length	Horizontal length between the front prosthion and nasion
Neurocranial length	Horizontal length between the nasion and inion
Horizontal skull length	Horizontal length between the front prosthion and inion
Horizontal optic chiasm-inion length	Horizontal length between the optic chiasm and inion
Frontal length	Distance from the tip of the zygomatic process of the frontal bone to the frontolacrimal sutures
Lacrimal length	Distance from frontolacrimal sutures to the junction between the lacrimal and zygomatic bones
Malar length	Distance from the junction between the lacrimal and zygomatic bones to the tip of the frontal process of the zygomatic bones
Orbital vertical length	Perpendicular distance between the supraorbital and infraorbital margins of the orbit
Orbital horizontal width	Horizontal distance between the rostral and caudal margins of the orbital rim
Orbital depth	Distance between the optic foramen and midpoint of the rostral and caudal margins of the orbital rim
Orbital index	Orbital width/orbital length × 100
Orbital area	22/7 ab, where a and b are half the orbital length and width, respectively
Zygomatic process angle	Angle between the left and right zygomatic processes of the frontal bones with optic chiasm as the vertex
Frontal–lacrimal angle	Angle between the left and right orbital rims of the frontal-lacrimal junction with optic chiasm as the vertex
Lacrimal–zygomatic angle	Angle between the left and right orbital rims of the lacrimal–zygomatic junction with optic chiasm as the vertex
Frontal process angle	Angle between the left and right frontal processes of the zygomatic bones with optic chiasm as the vertex
Optic chiasm angle	Angle between the left and right optic canals
